# Current Treatment Algorithms for Patients with Metastatic Non-Small Cell, Non-Squamous Lung Cancer

**DOI:** 10.3389/fonc.2017.00038

**Published:** 2017-03-20

**Authors:** Barbara Melosky

**Affiliations:** ^1^Medical Oncology, British Columbia Cancer Agency, Vancouver Centre, Vancouver, BC, Canada

**Keywords:** metastatic non-squamous non-small cell lung cancer, systemic therapy, chemotherapy, targeted therapy, epidermal growth factor receptor, anaplastic lymphoma kinase, algorithm

## Abstract

The treatment paradigm for metastatic non-small cell, non-squamous lung cancer is continuously evolving due to new treatment options and our increasing knowledge of molecular signal pathways. As a result of treatments becoming more efficacious and more personalized, survival for selected groups of non-small cell lung cancer (NSCLC) patients is increasing. In this paper, three algorithms will be presented for treating patients with metastatic non-squamous, NSCLC. These include treatment algorithms for NSCLC patients whose tumors have *EGFR* mutations, *ALK* rearrangements, or wild-type/wild-type tumors. As the world of immunotherapy continues to evolve quickly, a future algorithm will also be presented.

## Introduction

The previous standard of care in metastatic non-small cell lung cancer (NSCLC) was to treat patients with a platinum doublet for four to six cycles and to offer second-line therapy upon progression (1).

The emergence of molecular tests allows us to tailor treatment strategies based on the presence of driver mutations. Patients who have genetic alterations to epidermal growth factor receptor (*EGFR*) and anaplastic lymphoma kinase (*ALK*) now benefit from targeted therapies in the first line and beyond. In patients with no known driver mutations, the efficacy of immunotherapy with checkpoint inhibitors has revolutionized treatment. This area is evolving rapidly.

As new treatment options emerge, algorithms must balance the need to give the best drugs first with ensuring that there are still beneficial options available for later. The treatment algorithms discussed in this paper are based on Canadian recommendations. Although other health authorities may have different therapeutics available, many basic principles apply.

This paper discusses treatments for patients with non-squamous histology only.

Tumor mutation testing allows us to divide patients into three groups: patients with *EGFR-*positive tumor mutations (10–30%) (2); patients with *ALK* rearrangements (4–7%) (2); and patients with tumors who either do not have *EGFR* or *ALK* mutations, or their mutation status is unknown. As mutation testing expands to include new targets including human epidermal growth factor receptor 2 (*HER2*), *BRAF, RET* and *MET* and effective treatments are found, the treatment algorithms will increase in complexity (3).

## EGFR Mutation Positive

### First-line Therapies: Tyrosine Kinase Inhibitors

**Figure 1:**
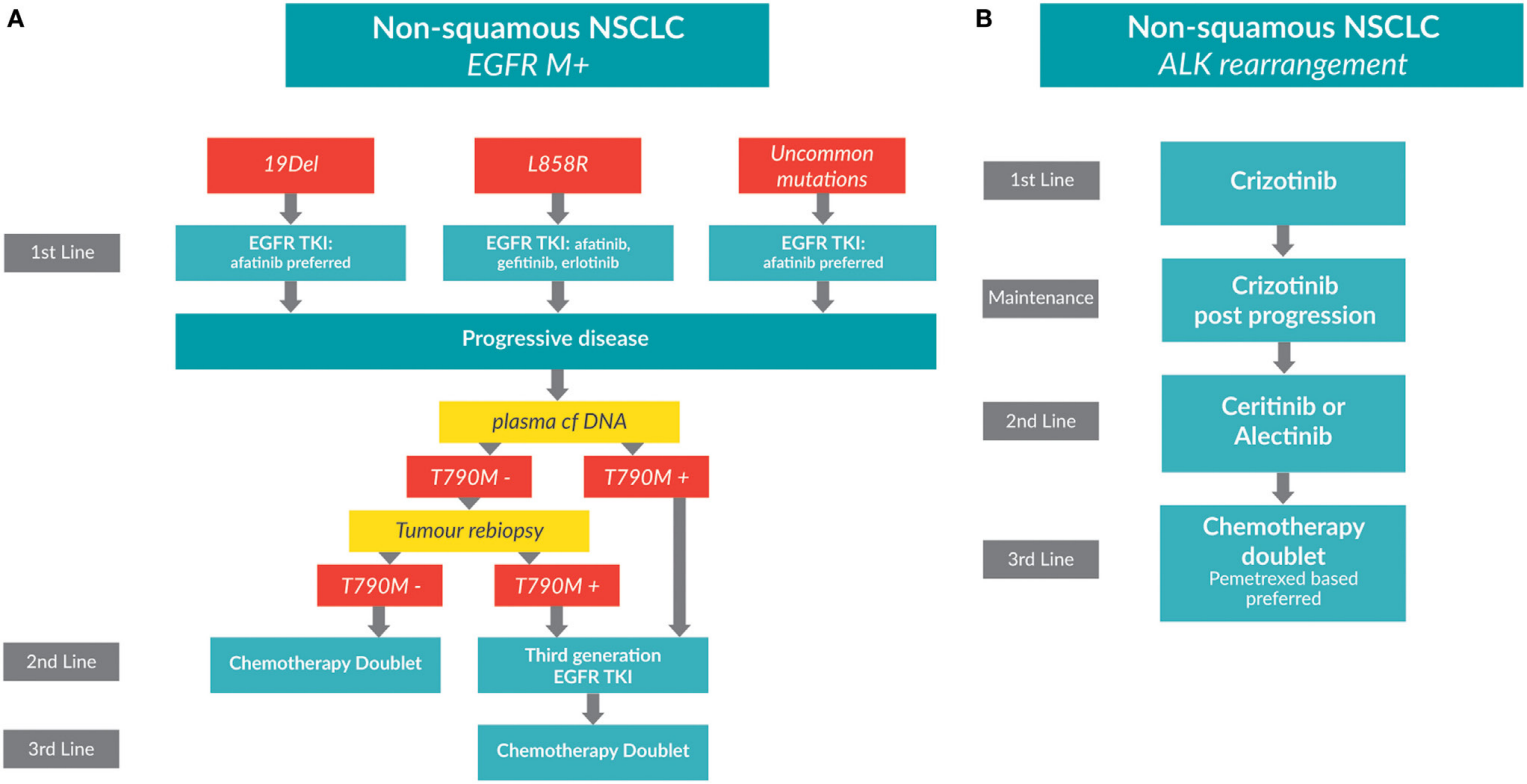
**Treatment algorithms for non-small cell lung cancer (NSCLC) patients whose tumors have driver mutations**. **(A)** A treatment algorithm for patients with *EGFR-*positive metastatic, non-squamous NSCLC [adapted from Melosky, Popat, and Gandara (submitted)]. **(B)** A treatment algorithm for patients with *ALK*-positive metastatic, non-squamous NSCLC.

Tyrosine kinase inhibitors (TKIs) that inhibit the EGFR are now standard of care for first-line treatment in patients with metastatic, non-squamous NSCLC whose tumors harbor an *EGFR* mutation (Figure 1A). Randomized trials have shown that patients experience superior overall response rates (ORR) and progression-free survival (PFS) when treated with EGFR TKIs versus chemotherapy for first-line therapy [erlotinib: EURTAC (4), OPTIMAL (5); gefitinib: NEJGSG_ 002 (6), WJTOG 3405 (7), IPASS (8, 9); afatinib: LUX LUNG 3 (10, 11), LUX LUNG 6 (11, 12)].

Erlotinib and gefitinib are first generation TKIs, while afatinib is a second generation TKI. Second generation TKIs block more ligands of the HER family and are non-competitive inhibitors at the kinase site so confer a longer period to resistance (13). Patient performance status, comorbidities, and age come into play in the decision making. EGFR mutation subtype is also important to consider. Unlike chemotherapy, TKIs are continued past progression as long as there is a clinical benefit to the patient.

LUX LUNG 7, a recently reported randomized phase IIb trial, compared afatinib to gefitinib in patients with advanced NSCLC and common *EGFR* mutations (14). The coprimary endpoint of PFS hazard ratio (HR) was met for superiority of afatinib, HR = 0.73 (*p* = 0.0165). This benefit was independent of mutation subtype. Response rate (RR), a secondary endpoint, was 70% versus 56% (HR = 1.873, *p* = 0.0083) favoring afatinib. Toxicities were as expected, with a preponderance of diarrhea and rash for afatinib and transaminitis for gefitinib. The overall survival (OS) was 3 months longer for afatinib (27.9 versus 24.5 months) but did not meet statistical significance [HR: 0.86 (95% CI: 0.66–1.12), *p* = 0.2580] (15). ARCHER 1050, a 452 patient phase III randomized trial of first-line treatment of *EGFR*-positive NSCLC comparing gefitinib with dacomitinib, will shed light on the question of which EGFR TKI is superior (16).

The inhibition of both EGFR and angiogenesis pathways deserves comment. The results of a randomized phase II trial from Japan illustrated a benefit for the combination erlotinib–bevacizumab over erlotinib for common EGFR mutations (17). Median PFS for the combination was 16.0 versus 9.0 months for erlotinib monotherapy, with no statistical difference in RRs or OS (18). In June 2016, the European Commission approved the combined use of erlotinib and bevacizumab for the first-line treatment of EGFR positive NSCLC patients. A larger phase III trial of this EGFR TKI–bevacizumab combination is needed to confirm and quantify the benefit (18).

### Retesting for EGFR Mutations on Progression

An acquired mutation in *EGFR* exon 20, *T790M*, which leads to drug resistance, may be found in up to 60% of patients progressing on TKIs (19). Repeat testing for mutations is now recommended. Testing plasma cell free (cf) DNA has been suggested as an alternative to repeat biopsy. Different testing platforms are being developed and validated, and concordance between cfDNA and tumor tissue is improving (20–23). Patients who initially test negative for the presence of a *T790M* mutation by cfDNA testing should undergo a tumor rebiopsy. Biopsy is still considered to be the gold standard for *T790M* molecular testing.

### Second-line Therapy

For patients with a *T790M* positive disease, third generation EGFR TKIs have demonstrated RRs of over 60% and prolonged PFS, resulting in the approval of osimertinib (AZD9291) in several countries. Pooled results from AURA phase I and II trials was recently presented, which evaluated osimertinib in patients with *T790M*-positive disease who progressed on previous EGFR TKIs (24). Patients from the pooled cohort (*n* = 411) had a RR of 66%, and a PFS of 11 months (24, 25).

For patients without a *T790M*, second-line therapy is a chemotherapy doublet. Patients who are *T790M* mutation negative who progress on chemotherapy have few other options and may consider a clinical trial.

## ALK Mutation Positive NCSLC

Rearrangements in the *ALK* gene are found in adenocarcinomas and more commonly in light or non-smokers. *ALK* rearrangements occur in approximately 4–7% of lung cancers (2). A treatment algorithm for patients with *ALK*-positive metastatic, non-squamous NSCLC is shown in Figure 1B.

### First-line Therapy with Crizotinib

For patients whose tumors are positive for an *ALK* rearrangement, crizotinib is superior to standard chemotherapy. The phase III PROFILE 1014 trial randomized 343 treatment-naïve patients with advanced *ALK* rearrangement positive NSCLC to receive either crizotinib or intravenous chemotherapy (26). The primary endpoint of PFS was significantly longer in patients treated with crizotinib at 10.9 months as compared to those treated with chemotherapy at 7.0 months [HR: 0.45 (95% CI: 0.35–0.60); *p* < 0.001]. Overall RRs were 74% for crizotinib and 45% for chemotherapy (*p* < 0.001). Median OS was not reached in either group due to cross-over [HR: 0.82 (95% CI: 0.54–1.26); *p* = 0.36] (26). Crizotinib was associated with a greater reduction in symptoms and better quality of life. As with other TKIs, crizotinib can be continued past progression if there is continuing clinical benefit to the patient.

### Second-line Therapy with Ceritinib, Alectinib, or Brigatinib

New agents are proving valuable as second-line treatments for NSCLC patients with *ALK*-positive tumors. As the brain is a frequent site of metastasis for patients with *ALK*-positive tumors, the intracranial activity of these agents is important to consider.

#### Ceritinib

Ceritinib is a second-generation ALK inhibitor that has demonstrated impressive RRs and has improved survival in patients who have progressed on crizotinib. The results of the ASCEND 1, 2, and 3 trials demonstrated the efficacy of ceritinib in treating both systemic disease and brain metastasis.

The ASCEND 1 phase I trial evaluated the efficacy and safety of ceritinib in 246 patients with advanced *ALK*-positive NSCLC (27). The ORR for all patients was 61.8%; 56.4% in pretreated patients and 72.3% in inhibitor-naïve patients. The PFS was 6.9 months for all trial participants (28). Of the 28 subjects with measurable brain metastases at baseline, 35% (*n* = 10) had a partial response (29). As a result of this trial, the FDA approved ceritinib for patients with advanced *ALK*-positive NSCLC following treatment with crizotinib in April 2014.

The ASCEND 2 single-arm phase II trial evaluated ceritinib efficacy in patients with advanced *ALK*-positive NSCLC who had progressed on both standard chemotherapy and crizotinib. With an ORR of 38.6% and a PFS of 5.7 months, ASCEND 2 confirmed the efficacy of ceritinib (30).

The ASCEND 3 single-arm phase II trial evaluated ceritinib efficacy in treatment-naïve patients with advanced *ALK*-positive NSCLC (31). In this trial, PFS was 11.1 months, with a RR of 36.3%. ASCEND 3 demonstrated that ceritinib has intercranial activity; a blinded independent central review demonstrated a 58.8% intracranial response in 50 (40.3%) of subjects with brain metastases (31).

#### Alectinib

Alectinib, another second-generation ALK inhibitor, also demonstrated impressive RRs and has improved survival in patients who have progressed on crizotinib. A phase I/II trial first evaluated the efficacy of alectinib in patients with crizotinib-refractory *ALK*-positive NSCLC; the dose determined in the phase I component was 600 mg orally twice a day (32). Two large phase II trials conducted in North America and internationally evaluated the efficacy and safety of alectinib in patients with *ALK*-positive NSCLC who had progressed on crizotinib. In the international study, an ORR of 50.8% was observed, the CNS ORR was 58.8% with 20.6% complete responses (33). In the North America trial, similar results were seen with an ORR of 52.2%. The CNS ORR in patients with measurable CNS metastases was 75%, with 25% complete responses (34). In both studies, Grade ≥ 3 adverse events were rare (33, 34).

Japanese researchers have studied alectinib in the first-line setting. The AF-001JP phase I study conducted in ALK treatment-naïve patients showed impressive efficacy. Because Japan has regulations on the use of sodium lauryl sulfate, the dose was set at just 300 mg twice daily (35).

Primary results of the phase III J-ALEX trial were presented at the 2016 American Society of Clinical Oncology meeting (36). In this trial, patients were randomized to receive either alectinib (300 mg twice a day) or crizotinib (250 mg twice a day) in the first-line setting. Alectinib demonstrated significant prolonged PFS [median PFS not reached (95% CI: 20.3 months–not estimated)] compared to crizotinib [PFS: 10.2 months (95% CI: 8.2–12.0)] (36). Although J-ALEX trial used a different dose than the global and North American trials, it led to FDA granting alectinib breakthrough therapy designation for first-line treatment (37).

#### Investigational Agents: Brigatinib and Lorlatinib

Brigatinib and the third-generation ALK inhibitor lorlatinib are being investigated for their efficacy and safety in *ALK*-positive NSCLC patients who have progressed after crizotinib and/or ceritinib (38, 39). Results of a phase II trial testing two doses of brigatinib demonstrated that patients who received the higher dose achieved a PFS of 12.9 months (40). As a result, the FDA gave brigatinib break through designation. Lorlatinib demonstrated efficacy in a phase I study in heavily pretreated patients; the ORR of 46% and a PFS of 11.4 months were impressive as most patients had received two or more lines of previous therapy (41). Both agents are active in CNS disease. We look forward to adding these agents to the algorithm.

#### ROS-1

The rare *ROS-1* rearrangement is now recognized as a standard biomaker in many countries, and several ALK inhibitors including crizotinib show activity in these patients. In May 2016, crizotinib was approved in the United States for patients with *ROS-1* rearranged NSCLC (42).

## Mutation Status Negative (“Wild-Type/Wild-Type”) NSCLC

### First-line Therapy: Platinum Doublet

**Figure 2:**
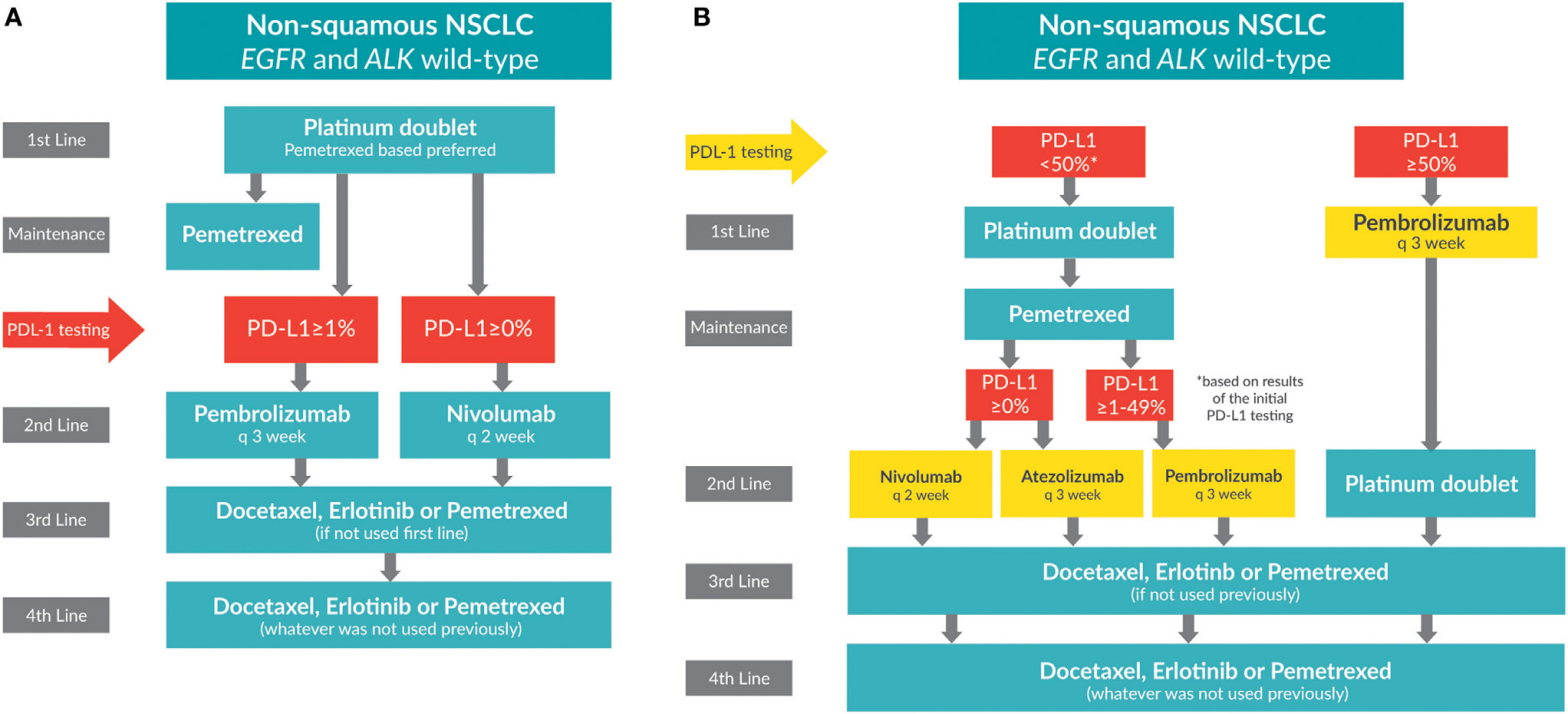
**Treatment algorithms for non-small cell lung cancer patients whose tumors do not have *EGFR* or *ALK* mutations (wild-type)**. **(A)** Current treatment algorithm. **(B)** Future treatment algorithm.

Patients with advanced NSCLC whose tumors do not have *EGFR* mutations or *ALK* rearrangements, or who have unknown mutation status, receive the standard of care: a platinum doublet (pemetrexed-based preferred) for four to six cycles (see Figure 2A). The Scagliotti trial demonstrated that NSCLC patients with adenocarcinoma experience greater benefit when treated with cisplatin/pemetrexed than with cisplatin/gemcitabine in the first line [OS: 12.6 versus 10.9 months; HR: 0.84 (95% CI: 0.71–0.99); *p* = 0.033] (1, 43).

### Maintenance Therapy

Maintenance therapy is administered after completion of first-line therapy but before disease progression. The PARAMOUNT trial demonstrated that pemetrexed maintenance after first-line chemotherapy significantly reduced disease progression over placebo for patients with non-squamous tumor histology (44). Studies have shown that pemetrexed improves both PFS and OS when administered as maintenance therapy (45). Although erlotinib was also an accepted option for switch maintenance based on the SATURN trial (46), the IUNO trial (HR: 1.02; 95% CI: 0.85–1.22; *p* = 0.82) did not support these results. As a consequence, erlotinib is no longer considered as a maintenance option for people with negative or unknown tumor mutation status (47).

### Second-line Therapy: Immune Checkpoint Inhibitors

The most important change in the NSCLC treatment paradigm has been the introduction and success of PD-L1 immune checkpoint inhibitors. The programmed cell death receptor (PD-1) is an inhibitory receptor on T lymphocytes that binds PD-L1 and PD-L2 ligands. When ligands PD-L1 and PD-L2 bind, the immune response is suppressed. PD-L1 overexpression by tumor cells allows them to escape T cell detection. Monoclonal antibodies targeting PD-1 or PD-L1 can lead to reactivation of the T lymphocyte and stimulate the natural immune response against tumor cells. Patients tested for PD-L1 overexpression can be categorized into PD-L1 expressers (≥1% expression) and non-expressers (<1% expression).

Trials evaluating three immunotherapy agents targeting the PD-1 pathway in NSCLC patients have demonstrated durable clinical activity and manageable toxicity (48–51).

#### Pembrolizumab

The KEYNOTE 010 trial compared pembrolizumab, a PD-1 monoclonal antibody, to docetaxel in the second-line NSCLC setting. The trial was positive for OS, favoring pembrolizumab at 10.4 months as compared to docetaxel at 8.5 months (HR: 0.71; *p* = 0.0008) (52). This trial included only patients whose tumors tested positive (> 1%) for the biomarker PD-L1. Pembrolizumab is administered intravenously every 3 weeks.

#### Nivolumab

Nivolumab, a monoclonal antibody against PD-1, was the first checkpoint inhibitor to show efficacy in a randomized phase III trial. The CHECKMATE 057 randomized phase III trial compared the efficacy of nivolumab with docetaxel as second-line treatment for patients with non-squamous NSCLC. Results showed OS benefits favoring nivolumab, at 12.2 months compared to 9.4 months for docetaxel (HR: 0.73; *p* = 0.0015) (53). Although survival was independent of whether the PD-L1 biomarker was present, there was a positive relationship between the degree of positivity of the biomarker and the level of benefit of the drug. Nivolumab is administered intravenously (3 mg/kg) every 2 weeks.

The decision about which antibody to use in the second line will depend on many factors. Determining the level of PD-L1 expression is complex. Biomarker testing and results, scheduling of drug administration (every 2 or every 3 weeks), cost, and availability all play a role.

### Third-Line Therapies and Beyond

Now that checkpoint inhibitors are used in the second line, the previously second-line therapies become third-line options for patients whose tumors are mutation negative or mutation unknown. Options include docetaxel (54), erlotinib (55), and pemetrexed (56). Pemetrexed can only be prescribed if it was not used in first line or maintenance therapy. The REVEL trial showed a benefit of adding the angiogenesis inhibitor ramucirumab to docetaxel, with PFS of 10.5 months for the combination versus 9.1 months for docetaxel alone (HR: 0.86; *p* = 0.23) (57).

It follows from above that fourth line therapies may include whatever agents were not administered in previous lines. A significant limitation of therapy selection is that no trials have tested these different agents in later lines of therapy. Patients with satisfactory performance status can be considered for clinical trials.

### Future Algorithm for Speculation Only

With many investigational agents in development, it is enticing to speculate what future treatment algorithms for patients with non-squamous NSCLC, mutation negative, or unknown mutation status (see Figure 2B).

#### High PD-L1 Expressers: Checkpoint Inhibitors in First-line Treatment

Recently, immune checkpoint inhibitors were tested in the first-line setting. The KEYNOTE O24 trial randomized patients whose tumors expressed >50% PD-L1 to pembrolizumab or a platinum doublet. The primary endpoint of PFS was met with 10.3 months favoring pembrolizumab, as compared to 6.0 months for chemotherapy (HR: 0.50; *p* < 0.001) (58). KEYNOTE 024 results will quickly be accepted due to the checkpoint inhibitor’s unique mechanism of action and low toxicity profile; we anticipate using pembrolizumab in the first line soon.

This contrasts with the results of the CHECKMATE 026 first-line trial of nivolumab, which randomized patients whose tumors expressed >5% PD-L1 to either nivolumab or a platinum doublet. The primary endpoint of PFS was not met, with 5.9 months favoring chemotherapy as compared to 4.2 months for nivolumab (HR: 1.15; *p* = 0.2511) (59).

For the high PD-L1 expressers, we speculate that second-line treatment will be a platinum doublet.

#### Low PD-L1 Expressers

In the future, patients who are low PD-L1 expressers will likely be treated with a platinum doublet in the first line. After progression, patients will be subdivided further based on the results of their initial PD-L1 test. In addition to pembrolizumab or nivolumab, many other agents are also in development.

#### Atezolizumab

Atezolizumab is a PD-L1 monoclonal antibody. The results of the OAK second-line trial comparing atezolizumab with docetaxel in patients with positive or negative PD-L1 expression were recently presented (60). The endpoint of OS was met, with results favoring atezolizumab at 13.8 months as compared to 9.6 months for docetaxel (HR: 0.73; *p* = 0.0003) (60). Atezolizumab is administered intravenously at a dose of 1200 mg/kg, every 3 weeks.

While speculating on the future of targeted therapy, will PD-1/PD-L1 checkpoint inhibitors will be prescribed for patients whose tumors are driven by *EGFR* mutations or *ALK* rearrangements? None of the immune therapy agents tested in the CHECKMATE 057 (59), KEYNOTE 010 (52), or OAK (60) trials showed efficacy in these patients. One reason for this may be because tumors with driver mutations have a low mutational load and low PD-L1 expression.

## Conclusion

Treatment algorithms for NSCLC have changed dramatically over the last few years. Researchers continue to elucidate many molecular pathways involved in thoracic malignancy. Our understanding of tumor mutations and their contribution to therapeutic efficacy is expanding. The treatment selection is complex, with many new target therapies being developed.

For patients with *EGFR*-driven tumors, treatment with osimertinib, a third-generation inhibitor, can lead to improvements in survival in patients whose tumors have acquired a *T790M* mutation. For patients with *ALK*-driven tumors who have progressed on crizotinib, new treatment options to improve survival include second-generation inhibitors ceritinib and alectinib. For patients without driver mutations or have an unknown tumor mutation status, chemotherapy remains the standard first-line treatment. The efficacy of checkpoint inhibitors has revolutionized treatment in the second-line setting; they now occupy the second-line setting and, on completion of KEYNOTE 024, we hope to see them in the first-line setting as well.

Targeted therapies are shifting the treatment paradigms and increasing survival for patients with NSCLC, a group that used to have a very poor prognosis. The ultimate winner is the patient.

## Author Contributions

BM wrote this article and created the algorithms. One algorithm is adapted from another paper (submitted).

## Conflict of Interest Statement

BM has received honoraria from Boehringer Ingelheim, Eli Lily, Pfizer, Roche, Merck, Bristol-Myers Squibb, Novartis, and AstraZeneca. She is in a consulting/advisory role with Boehringer Ingelheim, her institution has received research funding from Roche and Bayer, and she has received travel/accommodations/expenses from Boehringer Ingelheim, AstraZeneca, Novartis, and Pfizer.

## Abbreviations

ALK, anaplastic lymphoma kinase; EGFR, epidermal growth factor receptor; HR, hazard ratio; HER 2, human epidermal growth factor receptor 2; NSCLC, non-small cell lung cancer; OS, overall survival; ORR, overall response rate; RR, response rate; PFS, progression-free survival; TKI, tyrosine kinase inhibitor.
